# Hierarchical organization of multiscale communities in brain networks is non-tree structured

**DOI:** 10.1186/1471-2202-16-S1-P187

**Published:** 2015-12-18

**Authors:** Hiroshi Okamoto

**Affiliations:** 1 RIKEN Brain Science Institute, Saitama, 351-0198, Japan; 2 Research & Development Group, Fuji Xerox Co. Ltd., Kanagawa, 220-8668, Japan

## 

In literature of network science, a group of nodes that are densely connected within the group and are less connected with nodes outside the group is referred to as a "community" [1]. Community structure is a fundamental property of a variety of social, biological and engineering networks. Specifically, communities in brain networks are considered to be associated with functional modules of information processing in the brain [2]. To reveal information processing architecture of the brain, therefore, it is pivotal to know individual communities and their organization in brain networks.

Community structure in brain networks is characterized by hierarchical organization, which reflects that functional modules at larger scales are built up from a set of functional modules at smaller scales [3]. A number of mathematical methods for detecting communities in networks have been developed so far [1], but unfortunately few of them can consistently deal with hierarchical organization of multiscale communities. Here we propose a reliable method for detecting hierarchical organization of multiscale communities. Then we examine community structure of real brain networks by use of this method.

The proposed method is based on a novel Bayesian formulation of Markov chain. The method has only one parameter, , which comes from the precision of the prior distribution of a random process. The amplitude of controls the resolution of community detection; the smaller its amplitude, the finer the size of detected communities. Quasi-static increase in causes a series of discrete phase transitions; at each transition point a subset of smaller communities (children) agglomerate a larger community (parent), thus leading to a hierarchical organization of multiscale communities.

**Figure 1:**
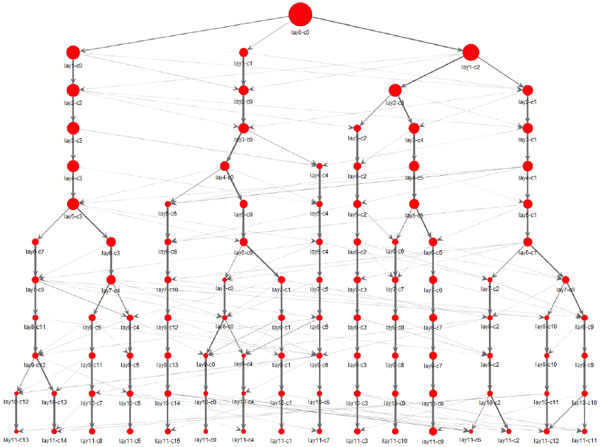
**Hierarchical organization of communities in the neuronal network of C**. elegans. Each layer of the hierarchy is shown by horizontal alignment of red squares. Each square indicates a community detected at each layer. The size of each square indicates the size of the corresponding community. Note that many communities have more than one link from the upper layer, which means that these communities have more than one parent. These demonstrate non-tree structure of hierarchical organization of communities in the brain network.

Applying this method to the neuronal network of C. elegans [4] and the macaque cortical network [5], we have found that hierarchical organization of multiscale communities in these networks is non-tree structured: Some child communities have more than one parent community (Figure 1). These findings suggest efficient architecture for integration of functional modules in brain information processing: The same functional modules at lower levels can be shared by distinct functional modules at higher levels.
